# Trends in Breast Augmentation Research: A Bibliometric Analysis

**DOI:** 10.1007/s00266-022-02904-9

**Published:** 2022-06-02

**Authors:** CholSik Ri, Jiang Yu, JiaXin Mao, MuXin Zhao

**Affiliations:** 1 The Second Affiliated Hospital of Dalian Medical University in China, Dalian, China; 2 The Pyongyang Medical University in D.P.R of Korea, Pyongyang, Democratic People’s Republic of Korea; 3 The Dalian Medical University in China, Dalian, China

**Keywords:** Breast augmentation, Augmentation mammaplasty, Breast implants, Bibliometric analysis

## Abstract

**Background:**

Breast augmentation is one of the most demanded procedures in plastic surgery and one of the most commonly performed by plastic surgeons. However, a bibliometric analysis of breast augmentation has not been published in recent years. The current study aimed to use a bibliometric analysis to conduct a qualitative and quantitative evaluation of breast augmentation research and provide the research trends and hotspots in this field.

**Methods:**

Publications on breast augmentation research were extracted from the Web of Science core collection database. VOSviewer 1.6.18 was used to assess co-authorship, co-occurrence, citation of countries, institutions, authors, and journals, as well as hotspot keywords.

**Results:**

On February 8, 2022, 4637 records of breast augmentation research published from 1985 to 2021 were collected. The bulk of the retrieved studies were original research articles (*n* = 2235, 48.20%). A total of 1053 (22.71%) papers were open access. The annual publication output increased annually. The USA was the driving force in this field and had a strong academic reputation. The top-contributing institution was the University of Texas MD Anderson Cancer Center (2.37%, with 110 publications). Plastic and reconstructive surgery (998 publications, 21.52%) published the most research in this field and was also the most frequently co-cited journal (22,351 citations, total link strength (TLS): 409,301). Clemens MW (68 publications, 1.47%) was the most prolific author, and Spear SL (1456 citations, TLS: 27,231) was the most frequently co-cited author. The research hotspots included the following four aspects: safety and effectiveness of breast implants, implant-based breast reconstruction, breast cancer incidence after breast implantation, and breast implant-associated anaplastic large-cell lymphoma (BIA-ALCL). The research trends were BIA-ALCL, implant-based breast reconstruction, BREAST-Q, acellular dermal matrix, capsular contracture, and autologous fat grafting.

**Conclusion:**

The present study provides a panoramic view of breast augmentation research in plastic and reconstructive surgery. This novel comprehensive bibliometric analysis can help researchers and nonresearchers alike to rapidly identify the potential partners, research hotspots, and research trends within their areas of interest.

**Level of Evidence III:**

This journal requires that authors assign a level of evidence to each article. For a full description of these Evidence-Based Medicine ratings, please refer to the Table of Contents or the online Instructions to Authors www.springer.com/00266.

## Introduction

Breast augmentation is one of the most demanded procedures in plastic surgery [1–5] and one of the most commonly performed procedures by plastic surgeons [6].

Reports of breast augmentation surgery date back to 1895, when a lipoma was successfully transplanted from the back to the breast defect in a mastectomy patient [6–9]. In the 1930s, implantation of a glass ball into a patient’s breast was the first implant-based breast augmentation [8]. The first modern breast implant was developed in 1961, and since then, there have been significant developments in implant composition and design [10].

Many reviews have summarized the clinical applications and experimental studies of breast augmentation in aesthetic plastic and reconstructive surgery [1, 5, 6, 10–13]. However, due to the rapid growth of scientific literature, it is difficult to generate a comprehensive assessment of this field. Manually compiling and systematically reviewing all publications in this field would be time-consuming, if not impossible.

Bibliometric analysis is a subject that quantitatively describes the current status, research hotspots, and trend information of science and technology based on the unique parameters of the published literature (such as countries, institutions, and authors), using a combination of mathematical and statistical methods. Both researchers and nonresearchers can use this information to quickly achieve specific aims in their area of interest (for example, to identify active partners, research topics, landmark documents, or other useful information) [14].

Bibliometric analysis was first applied by Pritchard in the 1960s and has been widely used in medicine and more recently in the plastic surgery literature [15–17].

In recent years, there have been many studies on breast augmentation, but a bibliometric analysis of breast augmentation has not been published recently. Therefore, we used a bibliometric analysis to conduct a qualitative and quantitative evaluation of breast augmentation research, and on this basis, we expect to identify emerging trends and hotspots in this field and predict future research priorities.

## Methods

The Web of Science core collection online database was queried with the following search string: Tl = (((breast augmentation)) OR (augmentation mammaplasty)) OR (breast implants)) OR (breast lipoaugmentation). Only original articles and reviews written in English and published from 1985 to 2021 were included.

Two investigators independently screened the literature, collected information, and cross-checked the references, and disagreements were resolved by a third author. VOSviewer 1.6.18 was used to assess the co-authorship, co-occurrence, citation of countries, institutions, authors, journals, and hotspot keywords. The size of the node represents the occurrence frequency of the relevant parameters, the thickness of the connection between nodes represents the degree of association, and the different colors represent different modules in the visualization mapping. The total link strength (TLS) attribute indicates the total strength of the co-authorship links of a given researcher with other researchers. The journal impact factor (IF) was obtained from the Journal Citation Reports by Thomson Reuters on January 30, 2021.

## Results

### Publication Output

**Fig. 1:**
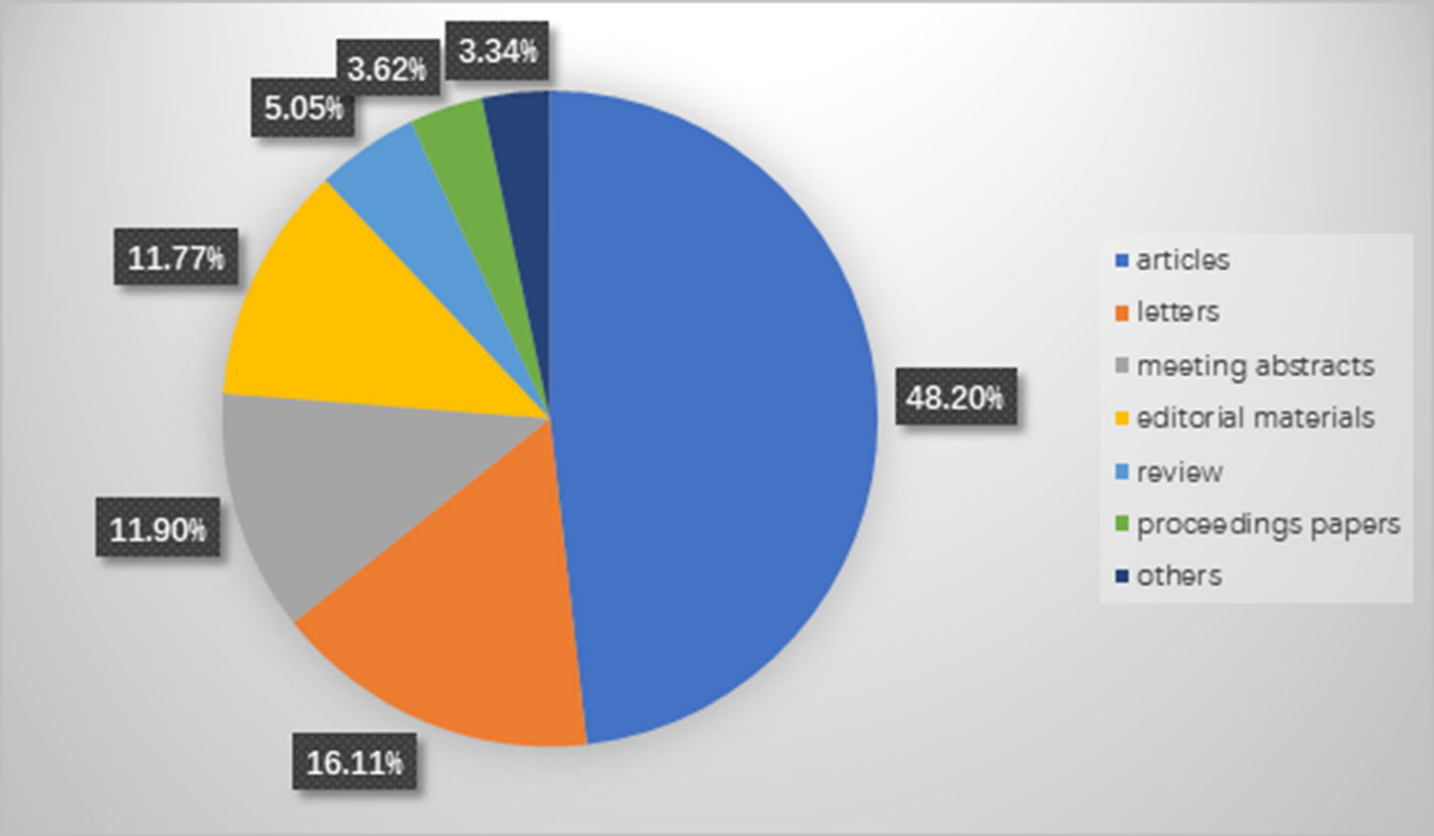
Study type composition summary

**Fig. 2:**
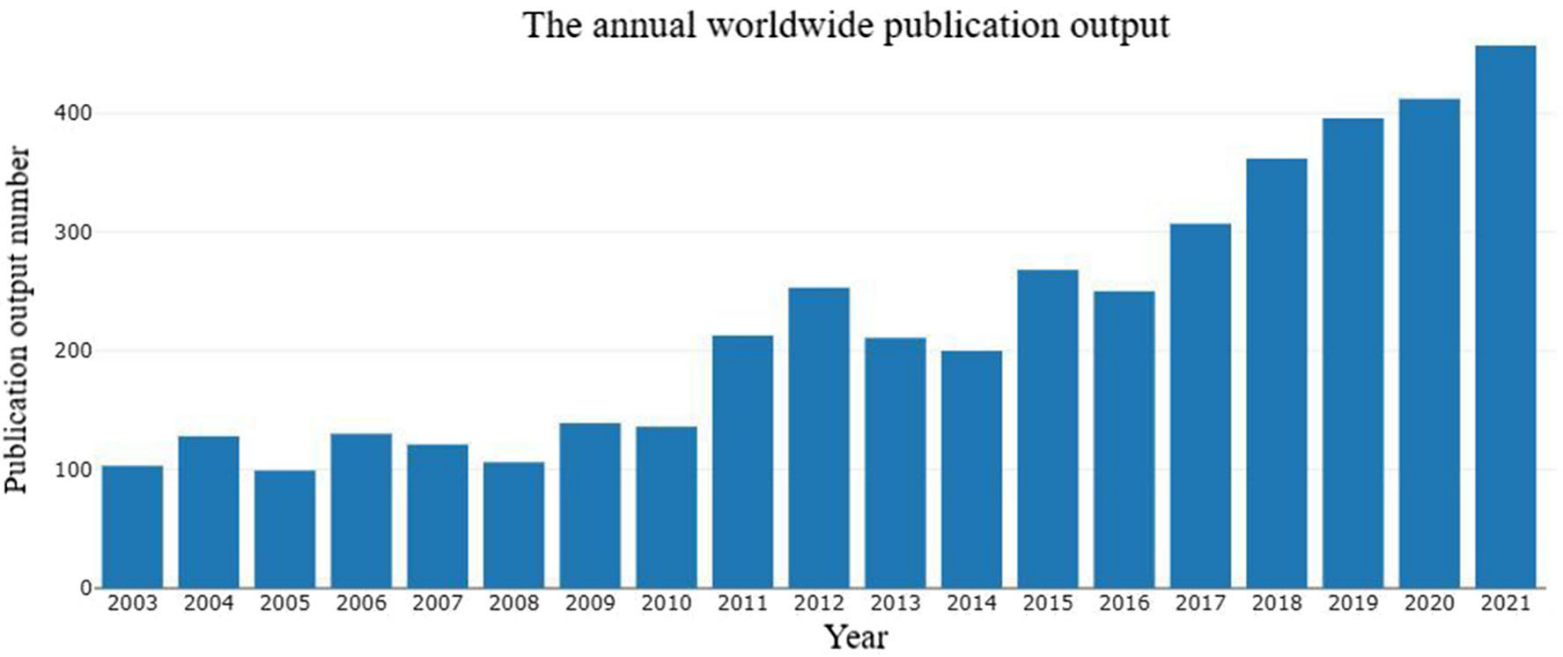
Annual worldwide publication output

From 1985 to 2021, a total of 4637 publications were identified from the Web of Science core collection online database (2022.2.8). These included 2235 (48.20%) original research articles, 747 (16.11%) letters, 552 (11.90%) meeting abstracts, 546 (11.77%) editorial materials, 234 (5.05%) review articles, 168 (3.62%) proceedings papers, and 155 (3.34%) other articles, including early access papers, among other publications (Fig. 1). A total of 1053 (22.71%) papers were open access. The growth of the annual publication output is shown in Fig. 2. Of these, 457 (9.86%) papers were published in 2021, and 412 (8.89%) papers were published in 2020. The annual publication output increased annually and increased to 457 in 2021 to more than two times the annual publication output in 2011.

### Analysis of Countries/Regions

**Fig. 3:**
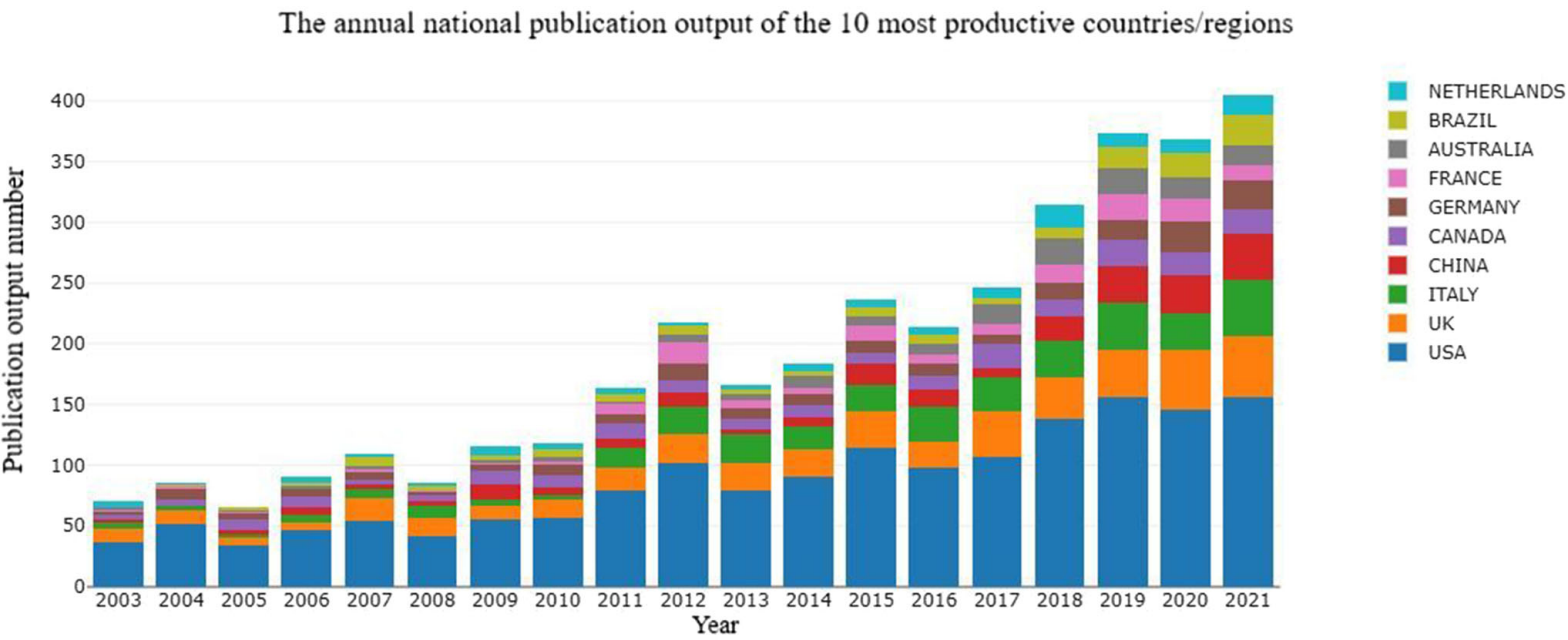
Annual national publication output of the ten most productive countries/regions

**Fig. 4:**
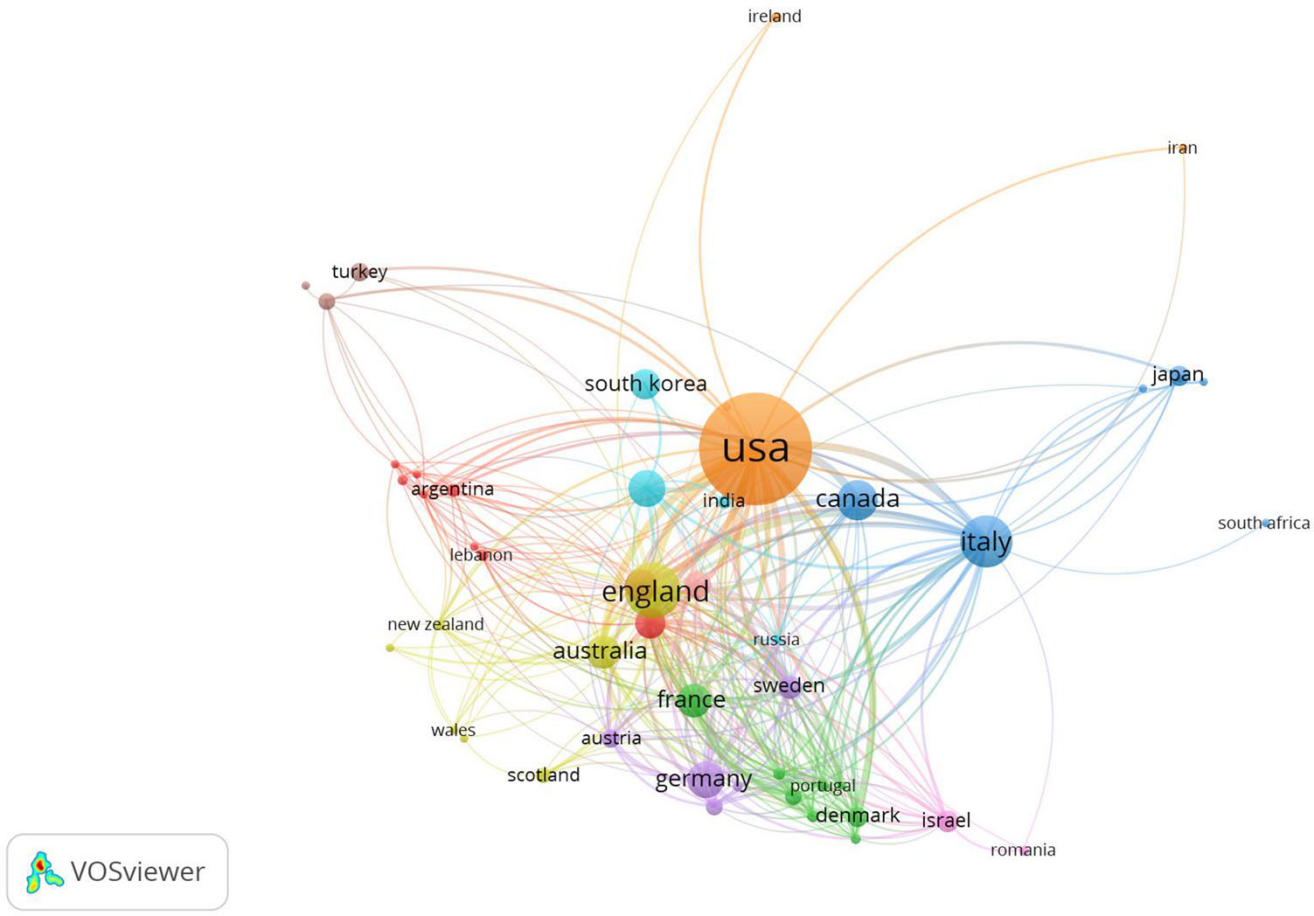
Network visualization map of country/regions related to breast augmentation research

All publications were distributed among 78 countries/regions. The annual national publication output of the ten most productive countries/regions is shown in Fig. 3. Since 2017, the annual growth rate of publication output has increased in England, followed by China, Germany, and Brazil. The ten most productive countries/regions are shown in Table 1. The USA had the highest output, with 1682 publications (36.27% of 4637 publications), followed by England (9.08%, with 421 publications), Italy (7.85%, with 364 publications), Canada (4.72%, with 219 publications), China (4.27%, with 198 publications), and Germany (4.23%, with 196 publications). The international collaboration network analysis is shown in Fig. 4. The size of the node indicates the number of publications issued in a specific country/region, and lines represent the frequency of cooperation between countries. Ten clusters were obtained based on this information. As shown in Fig. 4, the USA was the main driving force with a high academic reputation in breast augmentation research, which was confirmed by the following characteristics: number of publications (1682), H-index value (81), number of partners (45), total number of citations (31099), and TLS (388). Brazil had the highest number of citations per publication (CPP: 28.47). The total number of research publications from other countries, such as England, Italy, Canada, Germany, France, and Australia, was relatively low. However, their publication output still reflected the considerable progress made by these countries in this field, which was closely related to their collaborations with the USA.

**Table 1:** Top ten productive countries/regions

Rank	Country/region	Records	Percentage (%)	*H* (%)-Index	Citations	Citations per publication	Total link strength (TLS)
1	USA	1682	36.27	81	31099	18.49	388
2	England	421	9.08	35	3952	9.39	219
3	Italy	364	7.85	30	3631	9.98	186
4	Canada	219	4.72	36	3398	15.52	85
5	China	198	4.27	21	1479	7.47	51
6	Germany	196	4.23	26	2366	12.07	120
7	Australia	155	3.34	28	3484	22.48	138
8	France	151	3.26	20	1701	11.26	104
9	Brazil	146	3.15	22	4156	28.47	64
10	Netherlands	131	2.23	30	2419	18.47	95

### Analysis of Institutions

All publications were distributed among 3450 institutions. The ten most productive institutions are shown in Table 2. The leading institutions were the University of Texas MD Anderson Cancer Center (2.37%, with 110 publications), Memorial Sloan Kettering Cancer Center (2.09%, with 97 publications), Mayo Clinic (1.34%, with 62 publications), University of Toronto (1.34%, with 62 publications), and Chinese Academy of Medical Sciences (1.21%, with 56 publications). Most of the ten most productive institutions came from the USA, showing the strong academic influence of the USA in this field. The University of Texas MD Anderson Cancer Center (USA) and Vanderbilt University (USA) had the highest H-index (30), and Vanderbilt University (USA) had the highest number of citations per publication (38.81), which showed that they published more high-quality publications and played a pivotal role in promoting the development of this field.

**Table 2:** Top ten productive institutions

Rank	Institution	Records	Percentage (%)	*H* (%)-Index	Citations per publication	TLS	Location
1	University of texas MD anderson cancer center	110	2.37	30	24.22	185	United States
2	Memorial Sloan Kettering Cancer Center	97	2.09	31	38.71	90	United States
3	Mayo Clinic	62	1.34	14	11.56	63	United States
4	University of Toronto	62	1.34	22	16.22	52	United States
5	Chinese Academy of Medical Sciences	56	1.21	14	7.26	15	China
6	University of Washington	51	1.1	10	8.09	29	United States
7	Vanderbilt University	49	1.06	30	38.81	124	United States
8	MedStar Georgetown University Hospital	44	0.95	23	25.94	16	United States
9	University of California, Los Angeles	43	0.93	24	18.24	35	United States
10	Macquarie University	38	0.82	17	34.37	74	Australia

**Fig. 5:**
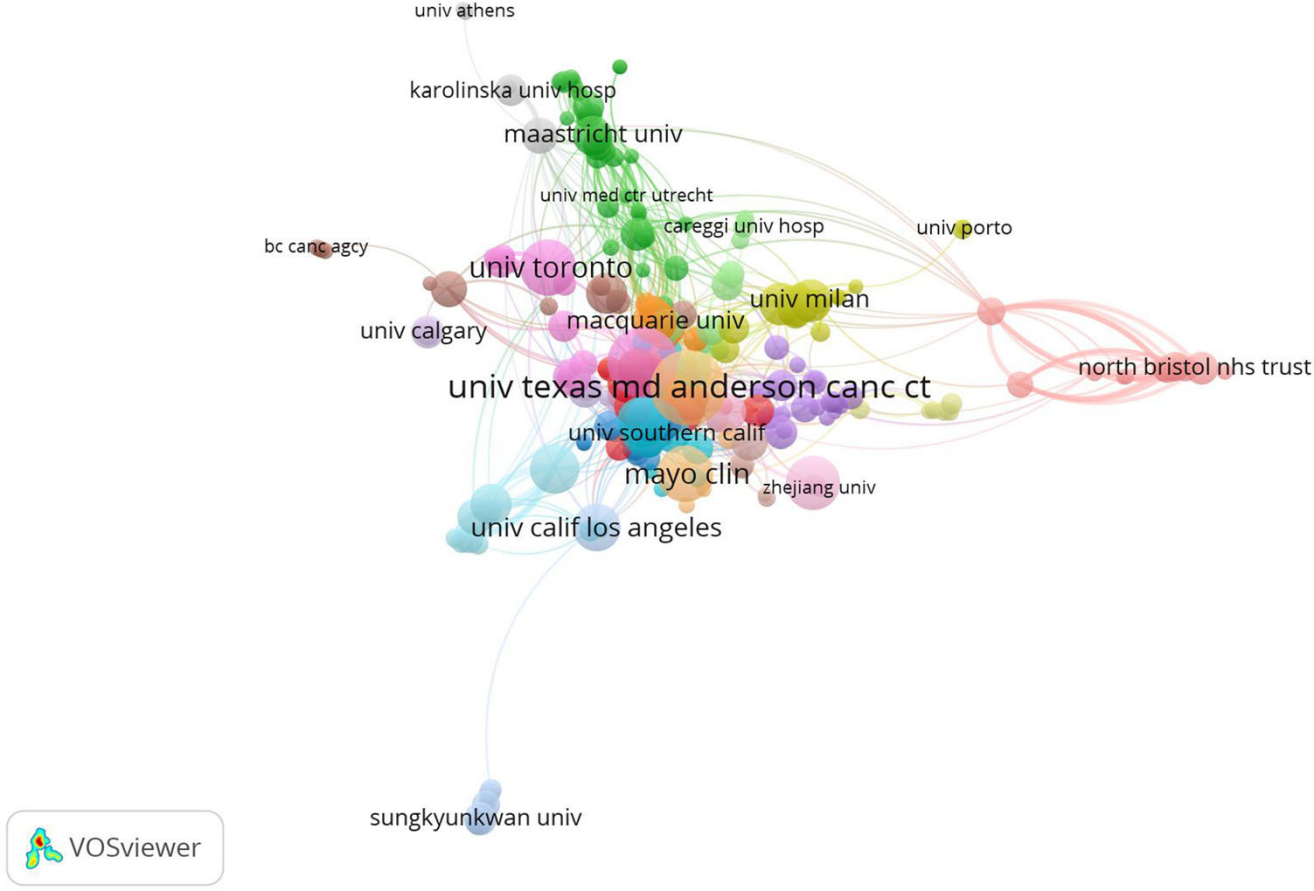
Network visualization map of institutions related to breast augmentation research

The network visualization map of cooperative institutions related to this research field is shown in Fig. 5. The nodes represent institutions, and lines between the nodes represent cooperative relationships. As shown in Fig. 5, the major institutions cooperative with the University of Texas MD Anderson Cancer Center were the Mayo Clinic, Memorial Sloan Kettering Cancer Center, Macquarie University, and University of Washington. Nineteen clusters were obtained from the analysis. For example, Cluster 1 (shown in red, 37 items) represented American academic institutions, such as Harvard University (links: 22, TLS: 30, publications: 34), New York University, University of Michigan, Loma Linda University, University of Pennsylvania, University of California San Francisco, and Northwestern University.

### Analysis of Journals and Co-cited Journals

**Fig. 6:**
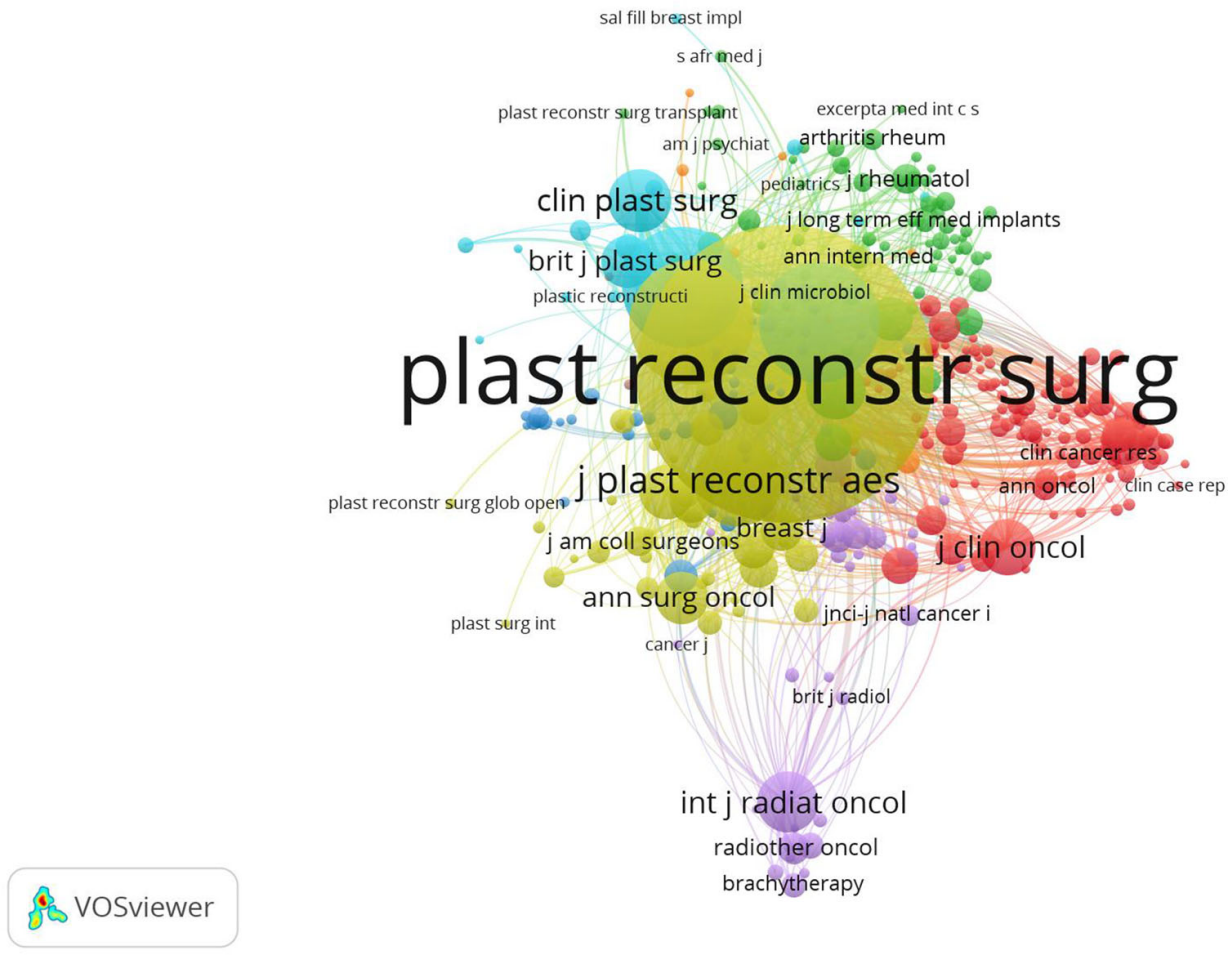
Network visualization map of co-cited journals related to breast augmentation research

The ten most productive and co-cited journals are listed in Tables 3, 4. A total of 520 journals published the relevant publications, of which 100 journals published more than five publications. In total, 2663 publications were published in the top ten active journals, which accounted for half of the publications on the Web of Science core collection online database. Plastic and reconstructive surgery (998 publications, 21.52%) published the most research in this field and had an IF of 4.73 in 2021, followed by Aesthetic Plastic Surgery (481 publications, 10.37%), Aesthetic Surgery Journal (391 publications, 8.43%), Journal of Plastic Reconstructive and Aesthetic Surgery (240 publications, 5.18%), and Annals of Plastic Surgery (233 publications, 5.02%). Among the top ten most productive journals, International Journal of Radiation Oncology Biology Physics had the highest IF (7.038), and plastic and reconstructive surgery had the highest number of citations per publication (21.3). Additionally, more than half of the top ten productive journals were classified in Q1 (the top 25% of the IF distribution). The most frequently co-cited journal was plastic and reconstructive surgery (22,351 citations, TLS: 409,301) (Fig. 6). The next most frequently co-cited journals were Annals of Plastic Surgery (3954 citations, TLS: 112,531), Aesthetic Surgery Journal (3689 citations, TLS: 99,091), and Journal of Plastic Reconstructive and Aesthetic Surgery (1944 citations, TLS: 54,254), showing that they were all important information resources. Among the top ten co-cited journals, New England Journal of Medicine had the highest IF (91.253), and plastic and reconstructive surgery had the highest H-index (70). More than half of the top ten co-cited journals were in Q1. Plastic and reconstructive surgery was recognized as a breast augmentation research resource and had an important influence on this research field.

**Table 3:** Top ten productive journals

Rank	Journal	Records	Percentage (%)	*I* (%) *F* (2021)	*H* (%)-Index	Citations per publication	Quartile in category
1	Plastic and Reconstructive Surgery	998	21.52	4.730	70	21.3	Q1
2	Aesthetic Plastic Surgery	481	10.37	2.326	32	9.75	Q3
3	Aesthetic Surgery Journal	391	8.43	4.283	30	10.19	Q1
4	Journal of Plastic Reconstructive and Aesthetic Surgery	240	5.18	2.740	29	11.58	Q2
5	Annals of Plastic Surgery	233	5.02	1.539	34	16.92	Q4
6	International Journal of Radiation Oncology Biology Physics	71	1.53	7.038	17	13.34	Q1
7	Breast Journal	69	1.49	2.431	16	11.93	Q3
8	Radiotherapy and Oncology	64	1.38	6.280	7	4.81	Q1
9	Annals of Surgical Oncology	60	1.29	5.344	12	8.42	Q1
10	British Journal of Surgery	56	1.21	6.939	6	4.2	Q1

**Table 4:** Top ten co-cited journals

Rank	Journal	Citations	TLS	*I* (%) *F* (2021)	*H* (%)-Index	Quartile in category
1	Plastic and Reconstructive Surgery	22351	409301	4.73	70	Q1
2	Annals of Plastic Surgery	3954	112531	1.539	34	Q4
3	Aesthetic Surgery Journal	3689	99091	4.283	30	Q1
4	Journal of Plastic Reconstructive and Aesthetic Surgery	1944	54254	2.74	29	Q2
5	Clinics in Plastic Surgery	1010	30749	2.017	15	Q3
6	International Journal of Radiation Oncology Biology Physics	961	20050	7.038	17	Q1
7	New England Journal of Medicine	893	28100	91.253	19	Q1
8	Journal of Clinical Oncology	797	24370	44.544	5	Q1
9	Annals of Surgical Oncology	723	21514	5.344	12	Q1
10	British Journal of Plastic Surgery	709	19393	1.291(2007)	6	Q2

### Analysis of Authors and Co-cited Authors

**Fig. 7:**
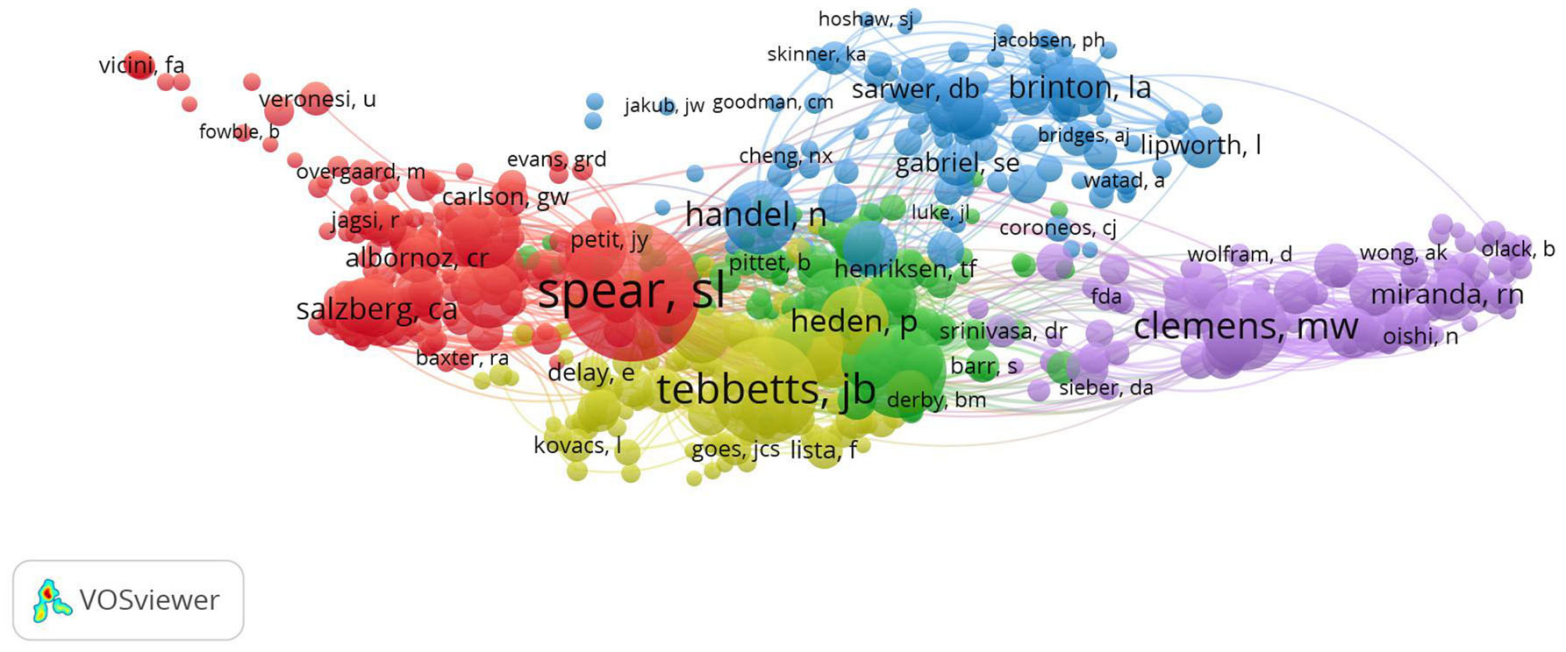
Network visualization map of co-cited authors related to breast augmentation research

A total of 13,320 authors have published papers on breast augmentation. The ten most productive authors and the ten top co-cited authors are shown in Table 5. Clemens MW (68 publications, 1.47%) published the most publications, followed by Adams WP (59 publications, 0.99%), Miranda RN (43 publications, 0.93%), Heden P (38 publications, 0.82%), Deva AK (36 publications, 0.78%), Medeiros IJ (33 publications, 0.71%), Pusik AL (27 publications, 0.58%), Cordeiro PG (26 publications, 0.56%), Maxwell GP (26 publications, 0.56%), and Mclaughlin JK (24 publications, 0.52%). The network visualization map of the co-cited authors is shown in Fig. 7. Five clusters were obtained from the analysis. The largest nodes were associated with the most frequently co-cited authors, including Spear SL (1,456 citations, TLS: 27,231, Cluster 1), Adams WP (653 citations, TLS: 14,432, Cluster 2), Handel N (404 citations, TLS: 8,941, Cluster 3), Tebbetts JB (874 citations, TLS: 13,323, Cluster 4), and Clemens MW (555 citations, TLS: 11,837, Cluster 5).

**Table 5:** Top ten productive authors and co-cited authors

Rank	Author	Records	Percentage (%)	Rank	Co-cited author	Citation	TLS
1	Clemens MW	68	1.47	1	Spear SL	1456	27231
2	Adams WP	46	0.99	2	Tebbetts JB	874	13323
3	Miranda RN	43	0.93	3	Adams WP	653	14432
4	Heden P	38	0.82	4	Clemens MW	555	11837
5	Deva AK	36	0.78	5	Maxwell GP	458	10429
6	Medeiros IJ	33	0.71	6	Handel N	404	8941
7	Pusik AL	27	0.58	7	Heden P	362	7235
8	Cordeiro PG	26	0.56	8	Cordeiro PG	338	6327
9	Maxwell GP	26	0.56	9	Nahabedian M	338	6026
10	Mclaughlin JK	24	0.52	10	Mccarthy CM	288	5674

### Analysis of References

**Fig. 8:**
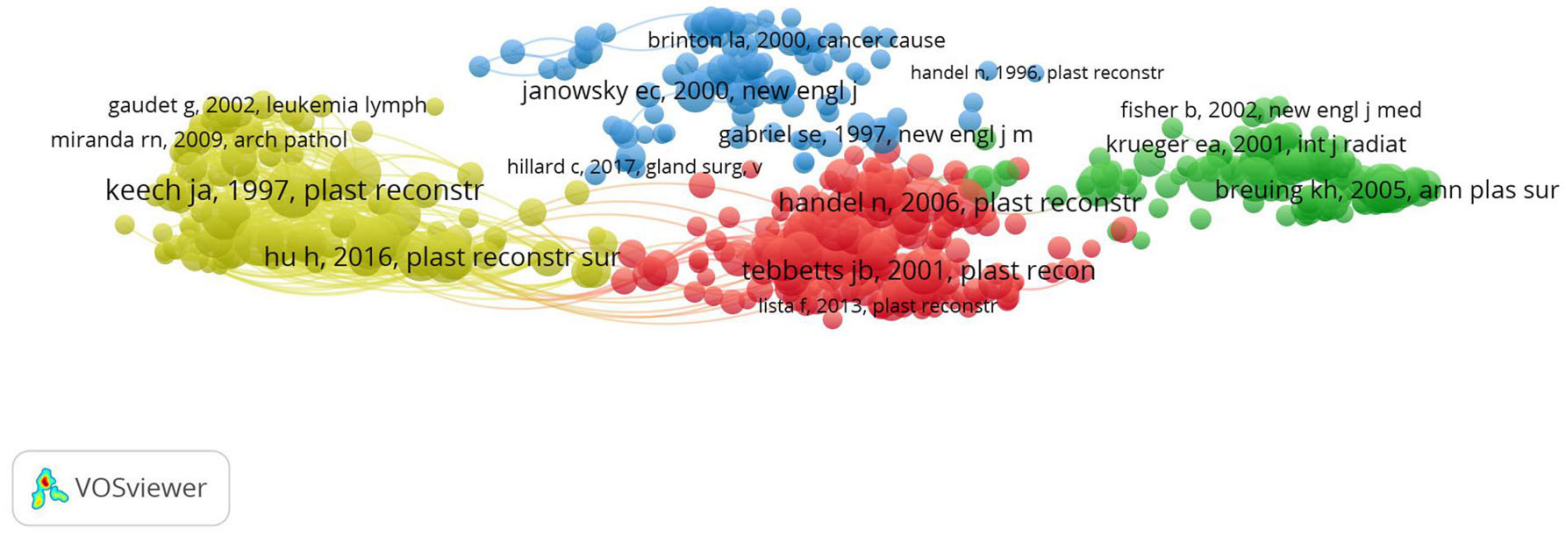
Network visualization map of co-cited references related to breast augmentation research

Figure 8 is a network map of co-cited references in this field, where there are 574 nodes and 49,315 links. According to Fig. 8, the size of the node represents the number of specific publications that have been cited. The more the literature is cited, the larger the diameter of the node. Table 6 summarizes the top 100 most-cited references according to the number of citations, including author, title, and year of publication. According to the 100 most-cited references, the title of “Anaplastic T-cell lymphoma in proximity to a saline-filled breast implant” published in Plastic and Reconstructive Surgery (IF 2021, 4.73) was the most-cited article, which was authored by Keech JA, with 705 citations, followed by “Yoshimura et al., 2008, Aesthetic Plast Surg”, “Albornoz et al., 2013, Plast Reconstr Sur”, “Breuing et al., 2005, Ann Plast Surg”, “Chun et al., 2010, Plast Reconstr Sur”, “Handel et al., 2006, Plast Reconstr Sur”, “McCarthy et al., 2008, Plast Reconstr Sur”, “Cordeiro et al., 2004, Plast Reconstr Sur”, “Yueh et al., 2010, Plast Reconstr Sur”, and “Pajkos et al., 2003, Plast Reconstr Sur”. Most of the top 100 most-cited references by citation count were in Q1. Most articles were published after 2005. We found that the research hotspots included the following four aspects: safety and effectiveness of breast implants (Cluster 1, red zone), implant-based breast reconstruction (Cluster 2, green zone), breast cancer incidence after breast implantation (Cluster 3, blue zone), and breast implant-associated anaplastic large-cell lymphoma (BIA-ALCL) (Cluster 4, yellow zone) (Fig. 8).

**Table 6:** Hundred most-cited references

Rank	Title	Journal IF (2021)	Author	Publication time	Citations	Quartile in category
1	Anaplastic T-cell lymphoma in proximity to a saline-filled breast implant	Plastic and Reconstructive Surgery (IF: 4.73)	Keech et al.	1997	705	Q1
2	Cell-assisted lipotransfer for cosmetic breast augmentation: Supportive use of adipose-derived stem/stromal cells	Aesthetic Plastic Surgery (IF: 2.326)	Yoshimura et al.	2008	603	Q3
3	A Paradigm Shift in US Breast Reconstruction: Increasing Implant Rates	Plastic and Reconstructive Surgery (IF: 4.73)	Albornoz et al.	2013	566	Q1
4	Immediate bilateral breast reconstruction with implants and inferolateral AlloDerm slings	Annals of Plastic Surgery (IF: 1.539)	Breuing et al.	2005	350	Q4
5	Implant-Based Breast Reconstruction Using Acellular Dermal Matrix and the Risk of Postoperative Complications	Plastic and Reconstructive Surgery (IF: 4.73)	Chun et al.	2010	344	Q1
6	A long-term study of outcomes, complications, and patient satisfaction with breast implants	Plastic and Reconstructive Surgery (IF: 4.73)	Handel et al.	2006	305	Q1
7	Predicting complications following expander/implant breast reconstruction: An outcomes analysis based on preoperative clinical risk	Plastic and Reconstructive Surgery (IF: 4.73)	McCarthy et al.	2008	300	Q1
8	Irradiation after immediate tissue expander/implant breast reconstruction: Outcomes, complications, aesthetic results, and satisfaction among 156 patients	Plastic and Reconstructive Surgery (IF: 4.73)	Cordeiro et al.	2004	256	Q1
9	Patient Satisfaction in Postmastectomy Breast Reconstruction: A Comparative Evaluation of DIEP, TRAM, Latissimus Flap, and Implant Techniques	Plastic and Reconstructive Surgery (IF: 4.73)	Yueh et al.	2010	254	Q1
10	Detection of subclinical infection in significant breast implant capsules	Plastic and Reconstructive Surgery (IF: 4.73)	Pajkos et al.	2003	247	Q1
11	Anaplastic large-cell lymphoma in women with breast implants	Jama-Journal of The American Medical Association (IF: 56.274)	De Jong D et al.	2008	245	Q1
12	Complete Surgical Excision Is Essential for the Management of Patients With Breast Implant-Associated Anaplastic Large-Cell Lymphoma	Clinical Oncology (IF: 4.126)	Clemens et al.	2016	239	Q3
13	A single surgeon's 12-year experience with tissue expander/implant breast reconstruction: Part I: A prospective analysis of early complications	Plastic and Reconstructive Surgery (IF: 4.73)	Cordeiro et al.	2006	236	Q1
14	Breast Implant-Associated Anaplastic Large-Cell Lymphoma: Long-Term Follow-Up of 60 Patients	Clinical Oncology (IF: 4.126)	Miranda et al.	2014	228	Q1
15	Implant-based breast reconstruction with allograft	Plastic and Reconstructive Surgery (IF: 4.73)	Zienowicz et al.	2007	225	Q1
16	Inferolateral AlloDerm hammock for implant coverage in breast reconstruction	Annals of Plastic Surgery (IF: 1.539)	Breuing et al.	2007	222	Q4
17	Infection in breast implants	Lancet Infectious Diseases (IF: 25.071)	Pittet et al.	2005	220	Q1
18	Textured Surface Breast Implants in the Prevention of Capsular Contracture among Breast Augmentation Patients: A Meta-Analysis of Randomized Controlled Trials	Plastic and Reconstructive Surgery (IF: 4.73)	Barnsley et al.	2006	213	Q1
19	Bacterial Biofilm Infection Detected in Breast Implant-Associated Anaplastic Large-Cell Lymphoma	Plastic and Reconstructive Surgery (IF: 4.73)	Hu et al.	2016	214	Q1
20	Anaplastic Large Cell Lymphoma Occurring in Women with Breast Implants: Analysis of 173 Cases	Plastic and Reconstructive Surgery (IF: 4.73)	Brody et al.	2015	211	Q1
21	Inamed silicone breast implant core study results at 6 years	Plastic and Reconstructive Surgery (IF: 4.73)	Spear et al.	2007	209	Q1
22	Breast Implant-Associated Anaplastic Large Cell Lymphoma in Australia and New Zealand: High-Surface-Area Textured Implants Are Associated with Increased Risk	Plastic and Reconstructive Surgery (IF: 4.73)	Loch-Wilkinson et al.	2017	201	Q1
23	*Brucella inopinata* sp nov., isolated from a breast implant infection	International Journal of System and Evolutionary Microbiology (IF: 2.747)	Scholz et al.	2010	201	Q3
24	The Effect of Acellular Dermal Matrix Use on Complication Rates in Tissue Expander/Implant Breast Reconstruction	Annals of Plastic Surgery (IF: 1.539)	Lanier et al.	2010	193	Q4
25	Capsular Contracture in Subglandular Breast Augmentation with Textured versus Smooth Breast Implants: A Systematic Review	Plastic and Reconstructive Surgery (IF: 4.73)	Wong et al.	2006	191	Q1
26	Comparison of Implant-Based Immediate Breast Reconstruction with and without Acellular Dermal Matrix	Plastic and Reconstructive Surgery (IF: 4.73)	Vardanian et al.	2011	191	Q1
27	Prepectoral Implant-Based Breast Reconstruction: Rationale, Indications, and Preliminary Results	Plastic and Reconstructive Surgery (IF: 4.73)	Sigalove et al.	2017	190	Q1
28	US Epidemiology of Breast Implant-Associated Anaplastic Large Cell Lymphoma	Plastic and Reconstructive Surgery (IF: 4.73)	Doren El	2017	188	Q1
29	Brava and Autologous Fat Transfer Is a Safe and Effective Breast Augmentation Alternative: Results of a 6-Year, 81-Patient, Prospective Multicenter Study	Plastic and Reconstructive Surgery (IF: 4.73)	Khouri et al.	2012	188	Q1
30	Implant reconstruction in breast cancer patients treated with radiation therapy	Plastic and Reconstructive Surgery (IF: 4.73)	Ascherman et al.	2006	179	Q1
31	Survival in breast cancer after nipple-sparing subcutaneous mastectomy and immediate reconstruction with implants: A prospective trial with 13 years median follow-up in 216 patients	Plastic and Reconstructive Surgery (IF: 4.73)	Benediktsson et al.	2008	179	Q1
32	Breast Implant-Associated Anaplastic Large Cell Lymphoma: A Systematic Review	Plastic and Reconstructive Surgery (IF: 4.73)	Gidengil et al.	2015	177	Q1
33	Subclinical (Biofilm) Infection Causes Capsular Contracture in a Porcine Model following Augmentation Mammaplasty	Plastic and Reconstructive Surgery (IF: 4.73)	Tamboto et al.	2010	169	Q1
34	Progenitor-Enriched Adipose Tissue Transplantation as Rescue for Breast Implant Complications	Breast Journal (IF: 2.431)	Yoshimura et al.	2010	165	Q3
35	Breast Implants and the Risk of Anaplastic Large-Cell Lymphoma in the Breast	Jama Oncology (IF: 31.777)	De Boer M et al.	2018	160	Q1
36	Five critical decisions in breast augmentation using five measurements in 5 minutes: The high five decision support process	Plastic and Reconstructive Surgery (IF: 4.73)	Tebbetts et al.	2005	160	Q1
37	Implant breast reconstruction using acellular dermal matrix	Annals of Plastic Surgery (IF: 1.539)	Gamboa-Bobadilla, GM	2006	157	Q4
38	Natrelle Round Silicone Breast Implants: Core Study Results at 10 Years	Plastic and Reconstructive Surgery (IF: 4.73)	Spear et al.	2014	155	Q1
39	Histological analysis of silicone breast implant capsules and correlation with capsular contracture	Biomaterials (IF: 12.479)	Siggelkow et al.	2003	153	Q1
40	Chronic Biofilm Infection in Breast Implants Is Associated with an Increased T-Cell Lymphocytic Infiltrate: Implications for Breast Implant-Associated Lymphoma	Plastic and Reconstructive Surgery (IF: 4.73)	Hu et al.	2015	149	Q1
41	Outcome of Different Timings of Radiotherapy in Implant-Based Breast Reconstructions	Plastic and Reconstructive Surgery (IF: 4.73)	Nava et al.	2011	149	Q1
42	Breast implant-associated anaplastic large cell lymphoma: two distinct clinicopathological variants with different outcomes	Annals of Oncology (IF: 32.976)	Laurent et al.	2016	143	Q1
43	Style 410 highly cohesive silicone breast implant core study results at 3 years	Plastic and Reconstructive Surgery (IF: 4.73)	Bengtson et al.	2007	139	Q1
44	Risk Factor Analysis for Capsular Contracture: A 5-Year Sientra Study Analysis Using Round, Smooth, and Textured implants for Breast Augmentation	Plastic and Reconstructive Surgery (IF: 4.73)	Stevens et al.	2013	139	Q1
45	Bacterial biofilms and capsular contracture in patients with breast implants	British Journal of Surgery (IF: 6.939)	Rieger et al.	2013	139	Q1
46	Breast Implant Complication Review: Double Capsules and Late Seromas	Plastic and Reconstructive Surgery (IF: 4.73)	Hall-Findlay, EJ	2011	136	Q1
47	Radiotherapy and immediate two-stage breast reconstruction with a tissue expander and implant: Complications and esthetic results	International Journal of Radiation Oncology Biology Physics (IF: 7.038)	Tallet et al.	2003	134	Q1
48	Surgical intervention and capsular contracture after breast augmentation - A prospective study of risk factors	Annals of Plastic Surgery (IF: 1.539)	Henriksen et al.	2005	133	Q4
49	Seroma-associated primary anaplastic large-cell lymphoma adjacent to breast implants: an indolent T-cell lymphoproliferative disorder	Modern Pathology (IF: 7.842)	Roden et al.	2008	132	Q1
50	A prospective assessment of surgical risk factors in 400 cases of skin-sparing mastectomy and immediate breast reconstruction with implants to establish selection criteria	Plastic and Reconstructive Surgery (IF: 4.73)	Woerdeman et al.	2007	132	Q1
51	Prepectoral implant placement and complete coverage with porcine acellular dermal matrix: A new technique for direct-to-implant breast reconstruction after nipple-sparing mastectomy	Journal of Plastic Reconstructive and Aesthetic Surgery (IF: 2.74)	Reitsamer et al.	2015	130	Q2
52	Cohesive silicone gel breast implants in aesthetic and reconstructive breast surgery	Plastic and Reconstructive Surgery (IF: 4.73)	Brown et al.	2005	128	Q1
53	The Mentor core study on silicone MemoryGel breast implants	Plastic and Reconstructive Surgery (IF: 4.73)	Cunningham et al.	2007	128	Q1
54	Irinotecan causes severe small intestinal damage, as well as colonic damage, in the rat with implanted breast cancer	Journal of Gastroenterology and Hepatology (IF: 4.029)	Gibson et al.	2003	127	Q2
55	Breast Reconstruction and Augmentation Using Pre-Expansion and Autologous Fat Transplantation	Clinics in Plastic Surgery (IF: 2.017)	Khouri et al.	2009	125	Q3
56	Anaplastic Large Cell Lymphoma and Breast Implants: A Systematic Review	Plastic and Reconstructive Surgery (IF: 4.73)	Kim et al.	2011	124	Q1
57	Dual plane breast augmentation: optimizing implant-soft-tissue relationships in a wide range of breast types	Plastic and Reconstructive Surgery (IF: 4.73)	Tebbetts et al.	2001	124	Q1
58	Development of a new patient-reported outcome measure for breast surgery: the BREAST-Q	Plastic and Reconstructive Surgery (IF: 4.73)	Pusic et al.	2009	123	Q1
59	The influence of radiotherapy on capsule formation and aesthetic outcome after immediate breast reconstruction using biodimensional anatomical expander implants	Journal of Plastic Reconstructive and Aesthetic Surgery (IF: 2.74)	Behranwala et al.	2006	122	Q2
60	NCCN Consensus Guidelines for the Diagnosis and Management of Breast Implant-Associated Anaplastic Large Cell Lymphoma	Aesthetic Surgery Journal (IF: 4.283)	Clemens et al.	2017	119	Q1
61	2019 NCCN Consensus Guidelines on the Diagnosis and Treatment of Breast Implant-Associated Anaplastic Large Cell Lymphoma (BIA-ALCL)	Aesthetic Surgery Journal (IF: 4.283)	Clemens et al.	2019	119	Q1
62	Subfascial breast implant: A new procedure	Plastic and Reconstructive Surgery (IF: 4.73)	Graf et al.	2003	119	Q1
63	Outcome Assessment of Breast Distortion Following Submuscular Breast Augmentation	Aesthetic Plastic Surgery (IF: 2.326)	Spear et al.	2009	116	Q3
64	Anaplastic Large Cell Lymphoma Associated With Breast Implants: A Report of 13 Cases	American Journal of Surgical Pathology (IF: 6.394)	Aladily et al.	2012	116	Q1
65	Primary breast augmentation clinical trial outcomes stratified by surgical incision, anatomical placement and implant device type	Journal of Plastic Reconstructive and Aesthetic Surgery (IF: 2.74)	Namnoum et al.	2013	115	Q2
66	Breast Augmentation Using Preexpansion and Autologous Fat Transplantation: A Clinical Radiographic Study	Plastic and Reconstructive Surgery (IF: 4.73)	Del Vecchio et al.	2011	115	Q1
67	Clinical and morphological conditions in capsular contracture formed around silicone breast implants	Plastic and Reconstructive Surgery (IF: 4.73)	Prantl et al.	2007	114	Q1
68	Cellular and molecular composition of fibrous capsules formed around silicone breast implants with special focus on local immune reactions	Journal of Autoimmunity (IF: 7.094)	Dolores et al.	2004	114	Q1
69	The mentor study on contour profile gel silicone MemoryGel breast implants	Plastic and Reconstructive Surgery (IF: 4.73)	Cunningham, B	2007	112	Q1
70	Natrelle Style 410 Form-Stable Silicone Breast Implants: Core Study Results at 6 Years	Aesthetic Surgery Journal (IF: 4.283)	Maxwell et al.	2012	111	Q1
71	Intracapsular allogenic dermal grafts for breast implant-related problems	Plastic and Reconstructive Surgery (IF: 4.73)	Baxter et al.	2003	110	Q1
72	Ten-Year Results From the Natrelle 410 Anatomical Form-Stable Silicone Breast Implant Core Study	Aesthetic Surgery Journal (IF: 4.283)	Maxwell et al.	2015	109	Q1
73	A Systematic Review of Complications of Implant-based Breast Reconstruction with Prereconstruction and Postreconstruction Radiotherapy	Annals of Surgical Oncology (IF: 5.344)	Momoh et al.	2014	109	Q1
74	Acellular Dermal Matrix for the Treatment and Prevention of Implant-Associated Breast Deformities	Plastic and Reconstructive Surgery (IF: 4.73)	Spear et al.	2011	108	Q1
75	US FDA Breast Implant Postapproval Studies Long-term Outcomes in 99,993 Patients	Annals of Surgery (IF:12.969)	Coroneos et al.	2019	107	Q1
76	The Process of Breast Augmentation: Four Sequential Steps for Optimizing Outcomes for Patients	Plastic and Reconstructive Surgery (IF: 4.73)	Adams, WP	2008	106	Q1
77	Implanted adipose progenitor cells as physicochemical regulators of breast cancer	Proceedings of the National Academy of Sciences of the United States of America (IF: 11.205)	Chandler et al.	2012	106	Q1
78	Classification of Capsular Contracture after Prosthetic Breast Reconstruction	Plastic and Reconstructive Surgery (IF: 4.73)	Spear et al.	1995	106	Q1
79	Breast Implant-Associated Anaplastic Large Cell Lymphoma A Systematic Review	Jama Surgery (IF: 14.766)	Leberfinger et al.	2017	105	Q1
80	The infected or exposed breast implant: Management and treatment strategies	Plastic and Reconstructive Surgery (IF: 4.73)	Spear et al.	2004	105	Q1
81	Benchmarking Outcomes in Plastic Surgery: National Complication Rates for Abdominoplasty and Breast Augmentation	Plastic and Reconstructive Surgery (IF: 4.73)	Alderman et al.	2009	104	Q1
82	Acellular Dermal Matrix-Assisted Direct-to-Implant Breast Reconstruction and Capsular Contracture: A 13-Year Experience	Plastic and Reconstructive Surgery (IF: 4.73)	Salzberg et al.	2016	104	Q1
83	Incidence of silicone breast implant rupture	Archives of Surgery (IF: 4.926)	Holmich et al.	2003	102	Q1
84	Macrotextured Breast Implants with Defined Steps to Minimize Bacterial Contamination around the Device: Experience in 42,000 Implants	Plastic and Reconstructive Surgery (IF: 4.73)	Adams et al.	2017	101	Q1
85	The Impact of Postmastectomy Radiotherapy on Two-Stage Implant Breast Reconstruction: An Analysis of Long-Term Surgical Outcomes, Aesthetic Results, and Satisfaction over 13 Years	Plastic and Reconstructive Surgery (IF: 4.73)	Cordeiro et al.	2014	101	Q1
86	Body image concerns of breast augmentation patients	Plastic and Reconstructive Surgery (IF: 4.73)	Sarwer et al.	2003	99	Q1
87	Complications in smokers after postmastectomy tissue expander/implant breast reconstruction	Annals of Plastic Surgery (IF: 1.539)	Goodwin et al.	2005	99	Q4
88	Effect of breast augmentation on the accuracy of mammography and cancer characteristics	Jama-Journal of the American Medical Association (IF: 56.274)	Miglioretti et al.	2004	99	Q1
89	Biomarkers Provide Clues to Early Events in the Pathogenesis of Breast Implant-Associated Anaplastic Large Cell Lymphoma	Aesthetic Surgery Journal (IF: 4.283)	Kadin et al.	2016	97	Q1
90	Treatment of Breast Animation Deformity in Implant-Based Reconstruction with Pocket Change to the Subcutaneous Position	Plastic and Reconstructive Surgery (IF: 4.73)	Hammond et al.	2015	96	Q1
91	A 15-Year Experience with Primary Breast Augmentation	Plastic and Reconstructive Surgery (IF: 4.73)	Codner et al.	2011	95	Q1
92	Patient Satisfaction With Postmastectomy Breast Reconstruction A Comparison of Saline and Silicone Implants	Cancer (IF:6.86)	McCarthy et al.	2010	95	Q1
93	Infection following Implant-Based Reconstruction in 1952 Consecutive Breast Reconstructions: Salvage Rates and Predictors of Success	Plastic and Reconstructive Surgery (IF: 4.73)	Reish et al.	2013	94	Q1
94	Factors that influence the decision to undergo cosmetic breast augmentation surgery	Journal of Womens Health and Gender-Based Medicine (IF: 2.111)	Didie et al.	2003	92	Q1
95	Style 410 cohesive silicone breast implant: Safety and effectiveness at 5 to 9 years after implantation	Plastic and Reconstructive Surgery (IF: 4.73)	Heden et al.	2006	92	Q1
96	Radiographic Findings after Breast Augmentation by Autologous Fat Transfer	Plastic and Reconstructive Surgery (IF: 4.73)	Veber et al.	2011	88	Q1
97	Fat Grafting and Breast Reconstruction with Implant: Another Option for Irradiated Breast Cancer Patients	Plastic and Reconstructive Surgery (IF: 4.73)	Salgarello et al.	2012	87	Q1
98	Long-term outcomes in breast cancer patients undergoing immediate 2-stage expander/implant reconstruction and postmastectomy radiation	Cancer (IF:6.86)	Ho et al.	2012	87	Q1
99	Pilot Study of Association of Bacteria on Breast Implants with Capsular Contracture	Journal of Clinical Microbiology (IF: 5.948)	Del Pozo et al.	2009	85	Q1
100	First report of a permanent breast Pd-103 seed implant as adjuvant radiation treatment for early-stage breast cancer	International Journal of Radiation Oncology Biology Physics (IF: 7.038)	Pignol et al.	2006	85	Q1

### Analysis of Author Keywords and Keyword Co-occurrence Clusters

**Fig. 9:**
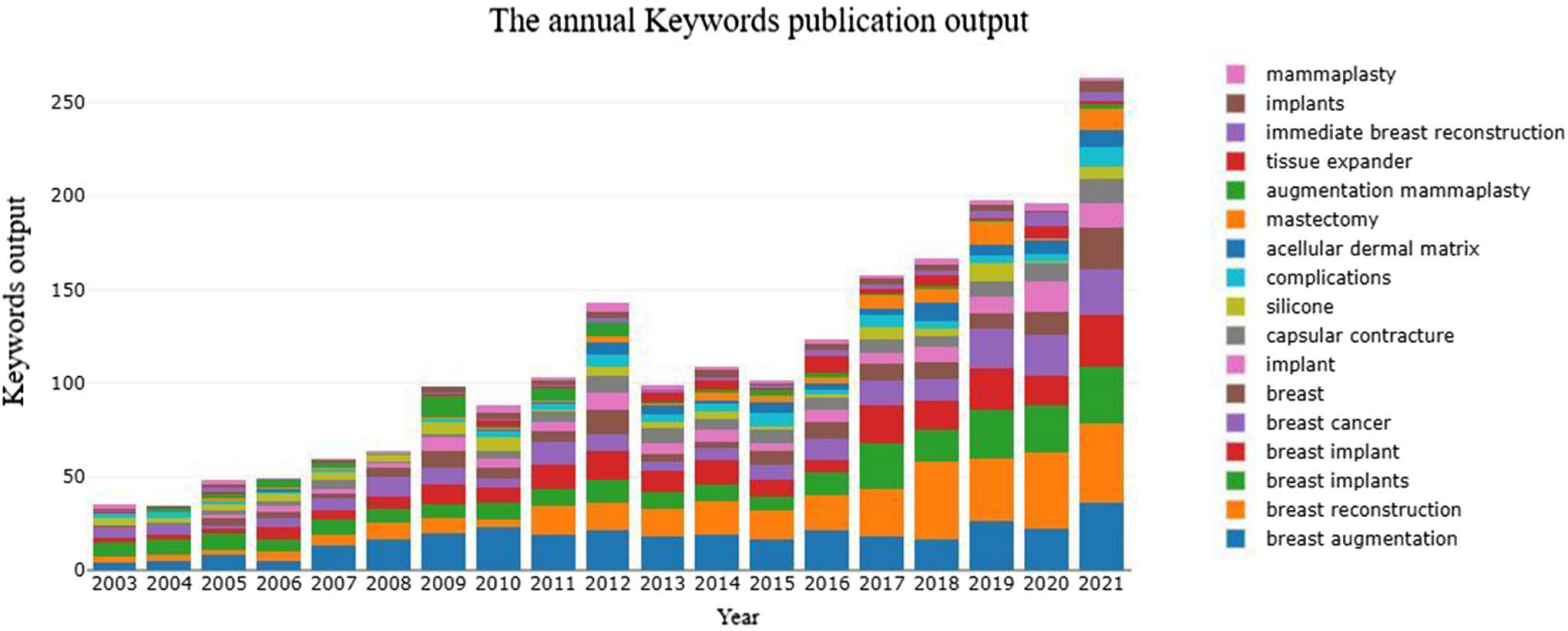
Annual keywords publication output of the breast augmentation research

**Fig. 10:**
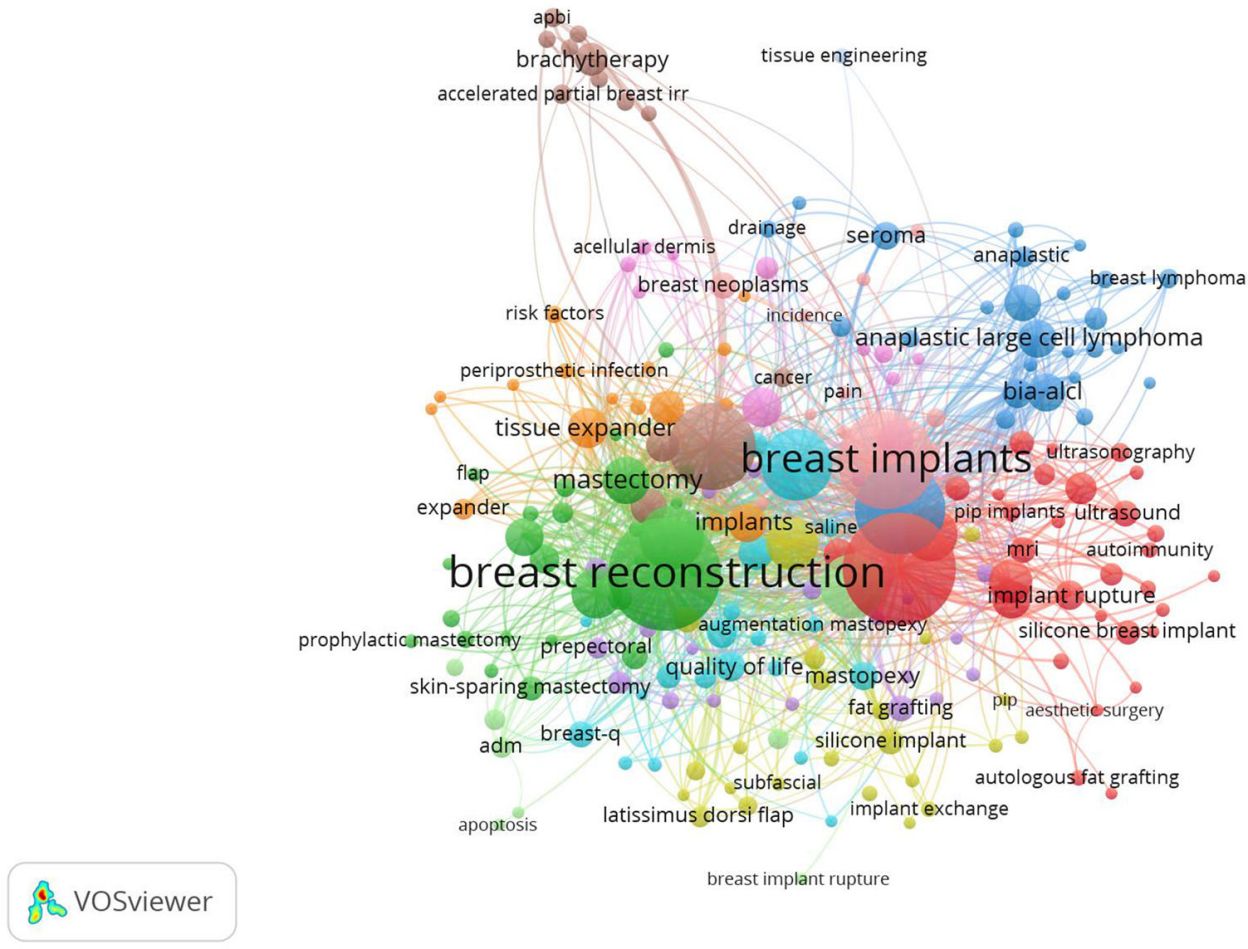
Cluster analysis of keyword co-occurrence

The annual keyword publication output of breast augmentation research is shown in Fig. 9. A total of 3316 keywords were considered in the analysis. Figure 10 shows a network map of keyword co-occurrence, where there are 210 items (occurrence >5) and 2253 links. Each item represents the frequency of a keyword appearing in the topic articles of our research. The larger the item is, the more clinical research on the subject surrounding the specific keyword. The thicker the line between the keywords, the higher the frequency of this group of keywords appearing in the clinical articles. The rapid identification of these keywords can help us quickly identify the hot topics studied in the literature. In addition, there are 12 clusters in total, and the name of each cluster is determined according to the number of items. After this analysis, we can further determine specific categories with an overview of this study derived from all keywords. The clinical significance of the co-occurrence keyword cluster map is that each cluster contains several different keywords for the subtheme of the study. Therefore, it can provide a convenient approach to help researchers who are concerned about the subtheme of this research field to quickly identify which other relevant keywords are contained in the subline they are concerned about from a global perspective.

According to the citation count analysis of keywords, we found that the most popular keywords were “breast reconstruction,” “breast augmentation,” “breast implants,” “breast implant,” “breast cancer,” “breast,” “implant,” “capsular contracture,” “silicone,” and “complications.” Cluster 1 (32 items) labeling “breast augmentation” (TLS: 577, occurrences: 333) was the largest cluster, followed by “breast reconstruction” (TLS: 792, occurrences: 335) (Cluster 2), “breast implant” (TLS: 451, occurrences: 216) (Cluster 3), “complications” (TLS: 223, occurrences: 75) (Cluster 4), “silicone implants” (TLS: 42, occurrences: 23) (Cluster 5), “breast” (TLS: 370, occurrences: 133) (Cluster 6), “tissue expander” (TLS: 141, occurrences: 45) (Cluster 7), “breast cancer” (TLS: 423, occurrences: 200) (Cluster 8), “mammaplasty” (TLS: 76, occurrences: 42) (Cluster 9), “breast implants” (TLS: 523, occurrences: 255) (Cluster 10), “capsular contracture” (TLS: 262, occurrences: 108) (Cluster 11), and “tissue engineering” (TLS: 7, occurrences: 7) (Cluster 12).

As shown in Fig. 9, “breast augmentation” was strongly associated with “breast reconstruction,” “breast implants,” “complications,” “silicone implants,” “breast,” “tissue expander,” “breast cancer,” “mammaplasty,” “capsular contracture,” “silicone,” and “tissue engineering.”

**Fig. 11:**
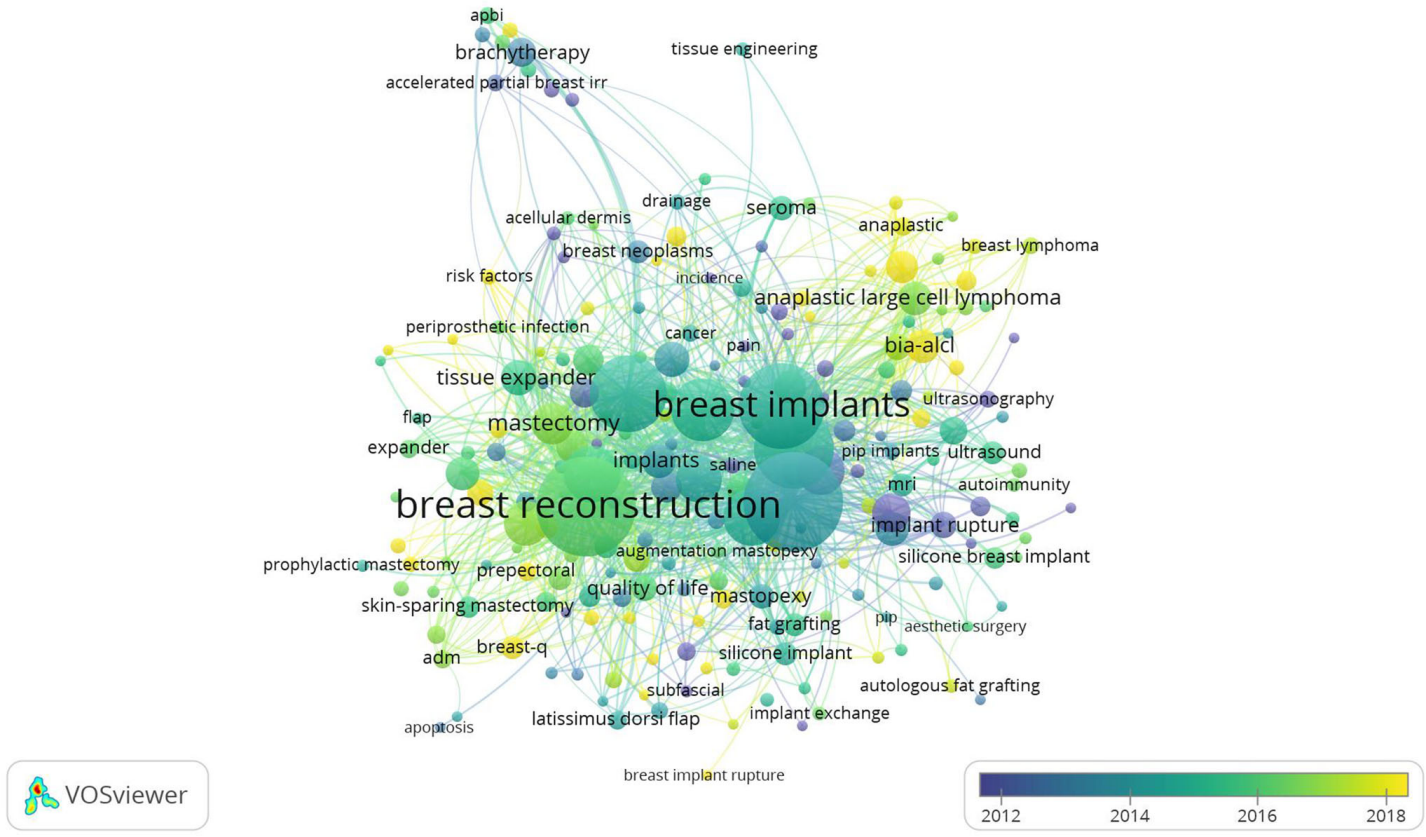
Timing analysis of keyword co-occurrence

Furthermore, several research directions, including “acellular dermal matrix (ADM)” (TLS: 170, occurrences: 67) (Cluster 2), “anaplastic large cell lymphoma” (TLS: 91, occurrences: 39) (Cluster 3), and “capsular contracture” (TLS: 262, occurrences: 108) (Cluster 11), have been the main topics since 2012 (Fig. 11).

The timing diagram showed that the research trends focused on breast implant-associated anaplastic large cell lymphoma (BIA-ALCL), implant-based breast reconstruction, and autologous fat grafting (Fig. 11).

## Discussion

Breast augmentation is one of the most commonly performed aesthetic surgeries every year [1]. This study identified 4637 publications related to breast augmentation research through the Web of Science core collection database from 1985 to 2021. This is probably the first bibliometric analysis in the available literature evaluating articles published on breast augmentation. During 2016–2021, the annual publication output increased steadily, and the USA was the main driving force with a high academic reputation in breast augmentation research. Furthermore, plastic and reconstructive surgery (USA) was recognized as a breast augmentation research resource and had an important influence on this research field.

### The Most-Contributing Authors and the Most-Cited References

Clemens MW from the University of Texas MD Anderson Cancer Center published the most publications (68 publications, 1.47%). His most-cited article mainly reported that surgical management with complete surgical resection is necessary to achieve optimal event-free survival in patients with BIA-ALCL [18].

Spear SL from the Department of Plastic Surgery at Georgetown University Hospital (USA) ranked first among all co-cited authors. His three most-cited articles focused on the safety and effectiveness of silicone breast implants and capsular contracture after prosthetic breast reconstruction [19–21]. These articles have been considered reliable reference resources for subsequent research.

Additionally, the paper entitled “Anaplastic T-cell lymphoma in proximity to a saline-filled breast implant” published in Plastic and Reconstructive Surgery was the most co-cited reference, which focused on the causal relationship between the presence of saline-filled breast implants and the development of non-Hodgkin’s lymphoma involving the breast. In this study, the researchers were involved in the care of a young woman with anaplastic T-cell lymphoma in proximity to saline-filled breast implants, in whom antineoplastic therapy was successfully accomplished without necessitating removal of the breast implants; the authors considered the presence of implants not to be an obstacle to the delivery of effective radiation therapy. A causal relationship between the presence of a saline-filled breast implant and the development of non-Hodgkin’s lymphoma involving the breast was not demonstrated [22]. Therefore, this article may provide insights for the study of the association of inflammatory or neoplastic diseases associated with saline-filled breast implants.

The second most-cited article was “Cell-assisted lipotransfer for cosmetic breast augmentation: supportive use of adipose-derived stem/stromal cells” published in Aesthetic Plastic Surgery. This article assessed the cell-assisted lipotransfer known as a strategy to overcome the problems with lipoinjection [23]. Lipoinjection is a promising treatment but has some problems, such as unpredictability and a low rate of graft survival due to partial necrosis. Yoshimura et al. used autologous adipose-derived stem cells in combination with lipoinjection and reported preliminary results suggesting that cell-assisted lipotransfer was effective and safe for soft tissue augmentation and superior to conventional lipoinjection [23].

The third most-cited article assessed long-term trends in rates and types of immediate breast reconstruction. Albornoz et al. suggested that the significant rise in immediate breast reconstruction rates in the USA correlated closely to an increase in implant use, and the reason for the increase in implant use was changes in mastectomy patterns, such as increased bilateral mastectomies [24].

### Research Hotspots

As shown in the network map of co-cited references (Fig. 8), the research hotspots included the following four aspects: safety and effectiveness of breast implants, implant-based breast reconstruction, breast cancer incidence after breast implantation, and breast implant-associated anaplastic large-cell lymphoma (BIA-ALCL).

“Safety and effectiveness of breast implants” (Cluster 1) included the most publications and indicated that the most common local complication was capsular contracture. In the sixth and tenth most-cited articles, Handel N et al. noted that the longer the implant was in place, the greater the cumulative risk of developing capsular contracture, and textured breast implants were better than smooth breast implants in decreasing the rate of capsular contracture [20, 21, 25–29].

“Implant-based breast reconstruction” (Cluster 2) indicated that the significant increase in immediate implant-based breast reconstruction rates was closely related to the expansion of implant use [19, 30]. The fourth, eighth, and ninth most-cited articles assessed implant-based reconstruction and additional options for this treatment [31–33].The fifth and seventh most-cited articles evaluated complications and clinical risk factors following implant breast reconstruction [34, 35].

“Breast cancer incidence after breast implantation” (Cluster 3) indicated the possibility that women who received implant-based breast augmentation for cosmetic purposes had an increased long-term risk of developing cancer. Janowsky EC et al. mentioned that there was no evidence of an association between silicone-gel-filled breast implants and any of the individual connective tissue diseases, all defined connective tissue diseases combined, or other autoimmune or rheumatic diseases [36, 37]. Brisson J et al. mentioned that women undergoing breast augmentation do not appear to have an increased long-term risk of developing cancer [38, 39].

“Breast implant-associated anaplastic large-cell lymphoma” (Cluster 4) showed that the association between breast implants and BIA-ALCL has been confirmed [22, 40–45]. The eleventh most-cited article by De Jong D in 2008 discussed whether the risk of anaplastic large T-cell lymphoma was associated with breast implants [41]. This article was seminal in this field, as evidenced by publication in JAMA-Journal of the American Medical Association, a high-impact factor medical journal. BIA-ALCL is a rare cancer in patients with breast implants, but the incidence is increasing [46, 47]. The nineteenth most-cited article by Hu H in 2016 identified bacterial biofilms in BIA-ALCL. Bacterial biofilms and a distinct microbiome found in BIA-ALCL samples suggest a possible cause of infection, and thus strategies to reduce their contamination should be more widely studied and practiced [42]. The twenty-second and twenty-eighth most-cited articles detailed that high-surface-area textured implants had been shown to significantly increase the risk of BIA-ALCL [43, 48]. The overall incidence of BIA-ALCL was 1.15 per 1000 textured implants and 1.79 per 1000 patients with textured implants, and the median time to diagnosis was 10.3 years (range, 6.4–15.5 years) [49]. The National Comprehensive Cancer Network (NCCN) guidelines on BIA-ALCL have been endorsed by the FDA and widely advocated by national professional societies, and the consensus guidelines have helped establish standardization of treatment for BIA-ALCL at all stages of the disease [50]. The treatment includes capsulectomy and implant removal at an early stage. However, systemic therapy is needed to include chemotherapy, radiation therapy (residual disease), and brentuximab vedotin at stages II to IV; complete capsular removal is the most important factor, and most patients can be cured [51].

### Research Trends

According to analysis of the most frequently used keywords and the timing diagram (Fig. 8), the research trends were as follows: breast implant-associated anaplastic large cell lymphoma (BIA-ALCL), implant-based breast reconstruction, BREAST-Q, acellular dermal matrix (ADM), capsular contracture, and autologous fat grafting.

The paper entitled “Prepectoral Implant-Based Breast Reconstruction: Rationale, Indications, and Preliminary Results” authored by Sigalove S and published in 2017 is the most frequently cited article associated with implant-based breast reconstruction among recently published publications. In this study, Sigalove S reported that by placing the implant prepectorally, problems related to pectoralis muscle elevation could be avoided [52]. Breast implant pocket locations are subglandular, subpectoral, subfascial, partially retropectoral, totally submuscular, and dual plane. Shen Z reported that the subfascial and subpectoral planes were safe and effective for controlling the overall complication rate and achieving a high satisfaction rate. The muscles of the chest, which are related to the optimal placement of implants, play an important role in breast augmentation surgery. The amount of muscle varies from patient to patient and may be a factor in determining whether submuscular or subglandular placement is best [53, 54]. Tebbetts JB mentioned that dual-plane augmentation mammaplasty adjusts the relationship between the implant and tissue to ensure adequate soft tissue coverage while optimizing implant-soft-tissue dynamics to offer more benefits than a single pocket location for various breast types [55].

The BREAST-Q can be used to study the impact and effectiveness of breast surgery from the patient’s perspective. The BREAST-Q has the potential to support advocacy, quality metrics, and an evidence-based approach to surgical practice by quantifying satisfaction and health-related quality of life [56].

Acellular dermal matrix (ADM) is developed from human skin (such as FlexHD, AlloMax, AlloDerm) or animal skin (such as SurgiMend), from which the cells are removed and the support structure is retained [57]. ADM has been widely used as an adjunct material for tissue-expander or implant-based breast reconstruction [34]. Implant-based breast reconstruction has been enhanced by ADM, and immediate single-stage direct-to-implant breast reconstruction with ADM may achieve aesthetic optimization by preserving the mastectomy skin envelope [30, 34]. Some researchers have demonstrated that human cadaveric skin-based products such as FlexHD, DermaMatrix, and ready-to-use AlloDerm have a similar risk of complications compared with those of freeze-dried AlloDerm, which has been used longer [57]. Another researcher mentioned that ADM was related to a greater risk of major complications from immediate implant-based breast reconstruction, especially in patients with a high BMI [58]. However, further investigation is needed.

Capsular contracture is a common complication of breast augmentation, and many techniques for prevention and treatment have been reported with inconsistent or variable results. Wagner DS mentioned that surgical capsectomy, prosthesis replacement, and acellular dermal matrix placement are effective methods for the treatment of capsular contracture after breast augmentation [59]. Some investigators suggest that adherence to a surgical technique focused on minimizing bacterial contamination of implants has greater clinical significance than implant surface characteristics when discussing capsular contracture. [60].

Autologous fat grafting for breast augmentation is a growing field, and the safety of this technique has been extensively evaluated [61]. Many authors have explored the effectiveness and safety of breast lipoaugmentation, focusing on volume change, fat retention, overall cosmetic improvement, and patient satisfaction [62]. Li FC mentioned that liposuction and autologous fat grafting were suitable approaches for breast augmentation [63]. Maione L described that the combination of high-profile round implants and fat grafting in breast augmentation can eliminate the risk of implant rotation to improve the aesthetic outcome and patient satisfaction [64, 65]. Cell-assisted lipotransfer (CAL) is a novel and promising technique for breast augmentation [66]. In cell-assisted lipotransfer, autologous adipose-derived stem (stromal) cells (ASCs) are used in combination with lipoinjection. This process converts relatively ASC-poor aspirated fat to ASC-enriched fat [67]. ASCs-enriched fat grafts had significantly higher retention rates than conventional fat grafts [68] and these results suggested that cell-assisted lipotransfer is superior to conventional lipotransfer for improving the fat survival rate in breast augmentation [69]. No serious or unexpected adverse events in autologous fat grafting for breast augmentation were reported, and all procedures were found to be safe and well-tolerated in all patients [70]. Time will prove whether these studies, as we mentioned above, will be real trends. There may be shortcomings from insufficient data or analysis, and more progressive studies are needed in the future.

## Conclusions

The highest number of publications was from the USA, followed by England, Italy, Canada, and China. The primary collaborators with the USA were England, Italy, Canada, Germany, France, and Australia.

The University of Texas MD Anderson Cancer Center and Spear SL from the Department of Plastic Surgery at Georgetown University Hospital (USA) were the most-cited institution and author, respectively. The most-cited journal was plastic and reconstructive surgery.

The research hotspots included the following four aspects: safety and effectiveness of breast implants, implant-based breast reconstruction, breast cancer incidence after breast implantation, and breast implant-associated anaplastic large cell lymphoma (BIA-ALCL).

The research trends were BIA-ALCL, implant-based breast reconstruction, BREAST-Q, acellular dermal matrix (ADM), capsular contracture, and autologous fat grafting.

This novel comprehensive bibliometric analysis of breast augmentation research can help researchers and nonresearchers alike to rapidly identify the potential partners, research hotspots, and research trends within their areas of interest.
